# Secondary hypoalbuminemia is independently associated with in-hospital mortality in children with *Escherichia coli* bloodstream infection

**DOI:** 10.1080/07853890.2026.2731669

**Published:** 2026-09-10

**Authors:** Jie Cheng, Ya Liu, Shaojun Li, Hongdong Li, Junming Huo, Liping Tan, Zhengxiu Luo

**Affiliations:** ^a^ Department of Emergency, Children’s Hospital of Chongqing Medical University, National Clinical Research Center for Children and Adolescents’ Health and Diseases, Ministry of Education Key Laboratory of Child Development and Disorders, Chongqing, China; ^b^ Intelligent Application of Big Data in Pediatrics Engineering Research Center of Chongqing Education Commission of China, Chongqing, China; ^c^ Chongqing Key Laboratory of Child Rare Diseases in Infection and Immunity, Chongqing, China; ^d^ Department of Pediatrics, Chongqing Youyoubaobei Women and Children^’^s Hospital, Chongqing, China; ^e^ Department of Intensive Care Unit, Children’s Hospital of Chongqing Medical University, National Clinical Research Center for Children and Adolescents’ Health and Diseases, Ministry of Education Key Laboratory of Child Development and Disorders, Chongqing, China; ^f^ Department of Respiratory Medicine, Children’s Hospital of Chongqing Medical University, National Clinical Research Center for Children and Adolescents’ Health and Diseases, Ministry of Education Key Laboratory of Child Development and Disorders, Chongqing, China

**Keywords:** Secondary hypoalbuminemia, mortality, *Escherichia coli*, Bloodstream infection, children

## Abstract

**Objective:**

Hypoalbuminemia is common in critically ill children, however, its prognostic role in pediatric *Escherichia coli* (*E. coli*) bloodstream infection (BSI) remains unclear. We aimed to evaluate the association between secondary hypoalbuminemia (hypoalbuminemia developing after BSI onset in patients with normal pre-infection albumin levels) and in-hospital mortality in children with *E. coli* BSI.

**Methods:**

We retrospectively enrolled 211 children with *E. coli* BSI. Logistic regression and Kaplan-Meier analysis were used to identify risk factors independently associated with in-hospital mortality. A Firth penalized regression was performed as sensitivity analysis.

**Results:**

In-hospital mortality was 12.32% (26/211). Secondary hypoalbuminemia after BSI occurred in 10.90% (23/211). Multivariable analysis identified four factors independently associated with in-hospital mortality: invasive mechanical ventilation (OR 46.40, 95% CI 3.90–552.62), secondary hypoalbuminemia (OR 10.59, 95% CI 1.39–80.91), higher PRISM III score (OR 1.18, 95% CI 1.03–1.36), and shorter time to positivity (TTP) (OR 0.71 per hour increase, 95% CI 0.56–0.89; i.e. shorter TTP was associated with higher mortality); all *p* < 0.05. Survival probability was significantly lower among children who developed secondary hypoalbuminemia (log-rank *p* = 0.002). Sensitivity analysis confirmed the robustness of these findings.

**Conclusions:**

Secondary hypoalbuminemia developing after *E. coli* BSI is independently associated with mortality in children. Secondary hypoalbuminemia may serve as a dynamic prognostic marker for increased in-hospital mortality in children with *E. coli* BSI. Prospective multicenter studies are warranted to determine its value for clinical risk stratification.

## Introduction

Bloodstream infection (BSI) remains a major global health problem associated with substantial morbidity and mortality [1]. The reported short-term mortality of BSI ranged from 10% to 30% [2], and the one-year mortality ranged from 8% to 48% [3]. BSI in children causes significant morbidity and mortality and leads to a high medical burden worldwide [4]. Despite advances in medical care, the burden of BSI in children remains substantial, underscoring the need for improved strategies to identify those at high risk of adverse outcomes [4], and more efforts should be made to improve outcomes in children with BSI. Children represent a vulnerable patient population, beyond prevention and early recognition of BSI, clinicians should prioritize prompt assessment of clinical severity and timely initiation of appropriate treatment [1]. Age-related physiological differences and the nonspecific presentation of severe infection in children may complicate early risk assessment and treatment decisions [5]. Hence, to help critically ill children receive intensive treatment promptly and avoid inappropriate treatment, there is a need for simple and readily available markers that can complement existing approaches to early risk stratification.

Several prognostic scoring systems with good performance have been reported, such as the Pediatric Risk of Mortality (PRISM) III scoring system [6], the Pediatric Index of Mortality (PIM) scoring system [6], the Pediatric Logistic Organ Dysfunction (PELOD) scoring system [6], and the Pediatric Sequential Organ Failure Assessment scoring (pSOFA) scoring system [7]. However, these scoring systems are sometimes cumbersome. Furthermore, time to positivity (TTP) [8], time to appropriate therapy (TTAT) [9], and other risk factors may independently correlate with mortality in children with BSI. Yet these indicators are limited by medical resources and take considerable time to obtain. As mentioned above, there is an urgent need to explore convenient and accessible indicators for children with BSI.

Serum albumin is a routinely measured and readily available biomarker that may provide additional prognostic information in patients with severe infection. Hypoalbuminemia is consistently associated with worse outcomes in critically ill adults and children, including prolonged stay and higher mortality [10–12]. In pediatric sepsis, low albumin levels predict adverse outcomes [13], and recent studies have shown that composite markers (e.g. ferritin-albumin ratio, lactate-albumin ratio) also have prognostic value [14,15]. In the specific setting of BSI, hypoalbuminemia has been identified as an independent risk factor for mortality in adults and in immunocompromised patients [16,17]. In patients with *E. coli* bacteraemia, the blood urea nitrogen-to-albumin ratio predicts 30-day mortality [14]. Recent multicenter data have further consolidated the prognostic value of albumin in pediatric critical care, demonstrating that admission hypoalbuminemia is associated with higher mortality, longer PICU stays, and increased need for organ support [18]. Serum albumin at emergency department admission has also been shown to predict 30-day mortality in infected patients, particularly in those with low-to-moderate illness severity [19]. Beyond absolute albumin levels, composite markers integrating albumin with other severity indicators, such as the lactate-to-albumin ratio (LAR), have shown promising prognostic performance. Elevated LAR has been independently associated with acute kidney injury and mortality in critically ill septic patients [20], and in pediatric nosocomial infections, LAR measured at 24 h demonstrated the highest predictive accuracy for mortality [21].

*Escherichia coli* (*E. coli*) is one of the most common bacterial pathogen causing BSI in children [4]. However, no study has specifically examined secondary hypoalbuminemia - i.e. hypoalbuminemia that develops after the onset of BSI in patients with normal pre-infection albumin levels. Unlike baseline hypoalbuminemia, secondary hypoalbuminemia may reflect dynamic changes associated with the systemic response to infection and therefore may provide prognostic information beyond admission albumin levels. Given the frequency of *E. coli* BSI in children and the potential value of a simple biomarker, we aimed to evaluate the prognostic value of serum albumin, with a focus on secondary hypoalbuminemia, in children with *E. coli* BSI. We hypothesized that secondary hypoalbuminemia would be independently associated with in-hospital mortality and could provide additional information for early clinical risk stratification.

## Methods

### Study designs and patients

This retrospective cohort study was conducted at the Children’s Hospital of Chongqing Medical University, a tertiary pediatric referral center in China. The requirement for informed consent was waived due to the study’s retrospective design. We enrolled inpatients (excluding newborns) with *E. coli* BSI from August 2015 to September 2021. Inclusion criteria were: (i) inpatients, excluding neonates; and (ii) positive blood culture for *E. coli*. Exclusion criteria were: (i) incomplete clinical records; (ii) polymicrobial BSI; or (iii) absence of appropriate antimicrobial therapy during hospitalization. The study did not involve any human or animal experiments and was approved by the Ethics Committee of the Children’s Hospital of Chongqing Medical University (Approval No. 2023-423). All methods adhered to the principles of the Declaration of Helsinki.

### Data collection and definitions

The collected data included demographic information, laboratory findings, imaging data, underlying conditions, diagnoses, treatments, complications, and outcomes. BSI was defined as the presence of positive blood cultures accompanied by systemic signs of infection [22], and the source of infection was determined according to the Centers for Disease Control and Prevention/National Healthcare Safety Network [23]. Nosocomial infection was defined as an infection occurring more than 48 h after admission [8]. Antimicrobial susceptibility breakpoints followed the Clinical and Laboratory Standards Institute criteria [24]. Bone marrow inhibition after chemotherapy was diagnosed according to the World Health Organization criteria [25]. Secondary hypoalbuminemia was defined as new-onset hypoalbuminemia occurring after BSI onset in patients whose albumin levels were within the age-specific normal range prior to infection. The date of first documented hypoalbuminemia was recorded as the exposure time. Patients with hypoalbuminemia on admission were not classified as having secondary hypoalbuminemia; they were included in the non-secondary hypoalbuminemia reference group. Serum albumin was measured at admission and thereafter according to clinical need rather than a fixed protocol, every 48–72 h in unstable patients or those with abnormal levels, and approximately weekly in stable patients. Hypoalbuminemia was defined using age-specific thresholds (< 25 g/L for infants < 7 months; < 34 g/L for children ≥7 months) [26]. The maximum interval from BSI onset to the first low albumin measurement was 72 h. TTP was defined as the interval between incubation of the blood culture specimen and the alert signal from the blood culture system [8]. TTAT was defined as the period from BSI identification to the initiation of appropriate antimicrobial treatment [27]. Serous cavity effusion was diagnosed by imaging (e.g. ultrasound, X-ray, and so on.) [28]. Polyserous effusions was defined as effusion involving at least two serous cavities [29]. Septic shock was defined as severe infection leading to cardiovascular dysfunction [30].

### Data source statement

This study is a secondary analysis of a pediatric *E. coli* BSI cohort at the Children’s Hospital of Chongqing Medical University. Parts of this cohort have been previously used to investigate risk factors for antibiotic resistance [31] and the effect of empirical carbapenem therapy [32]. The current study addresses a distinct research question regarding the prognostic role of hypoalbuminemia, which has not been explored in prior publications from this cohort.

### Statistical analysis

Continuous variables with a normal distribution were presented as mean ± standard deviation, while those with a non-normal distribution were presented as median (interquartile range, IQR). Categorical variables were expressed as numbers and percentages. Comparisons between groups were performed using Student’s t-test or Mann-Whitney U test for continuous variables, and the chi-square (χ^2^) test or Fisher’s exact test for categorical variables, as appropriate. Univariate logistic regression was used to identify potential risk factors for mortality. Variables with a P-value < 0.10 in the univariate analysis were entered into a multivariable logistic regression model to determine independent risk factors for mortality in children with *E. coli* BSI. Variables were first selected based on clinical plausibility and prior literature, with additional consideration of those showing *p* < 0.10 in univariate analysis. Survival differences between groups were compared using Kaplan-Meier curves with the log-rank test. Disease severity was assessed using the Pediatric Risk of Mortality (PRISM) III score [6]. Multicollinearity was assessed by Variance inflation factor (VIF) (threshold > 5 for removal). Subgroup analyses (leukemia, bone marrow inhibition after chemotherapy) were performed using univariate logistic regression with interaction tested by likelihood ratio test. A penalized logistic regression (Firth’s method) was conducted as a sensitivity analysis. To address potential immortal time bias, landmark analyses were performed at days 7, 14, 21, and 28 after BSI onset, using length of hospital stay as the time scale. Hazard ratios were estimated using Cox regression with remaining length of stay as the time axis. A time-varying coefficient Cox model was fitted to test whether the effect changed over time. Kaplan-Meier curves with log-rank tests were plotted for the overall cohort and each subgroup. ROC analysis was performed to evaluate the discriminatory ability of these four factors in combination for in-hospital mortality. All statistical analyses were performed using R software (version 4.4.1; R Foundation for Statistical Computing). A two-sided P-value < 0.05 was considered statistically significance.

## Results

### Study population

**Figure 1.**
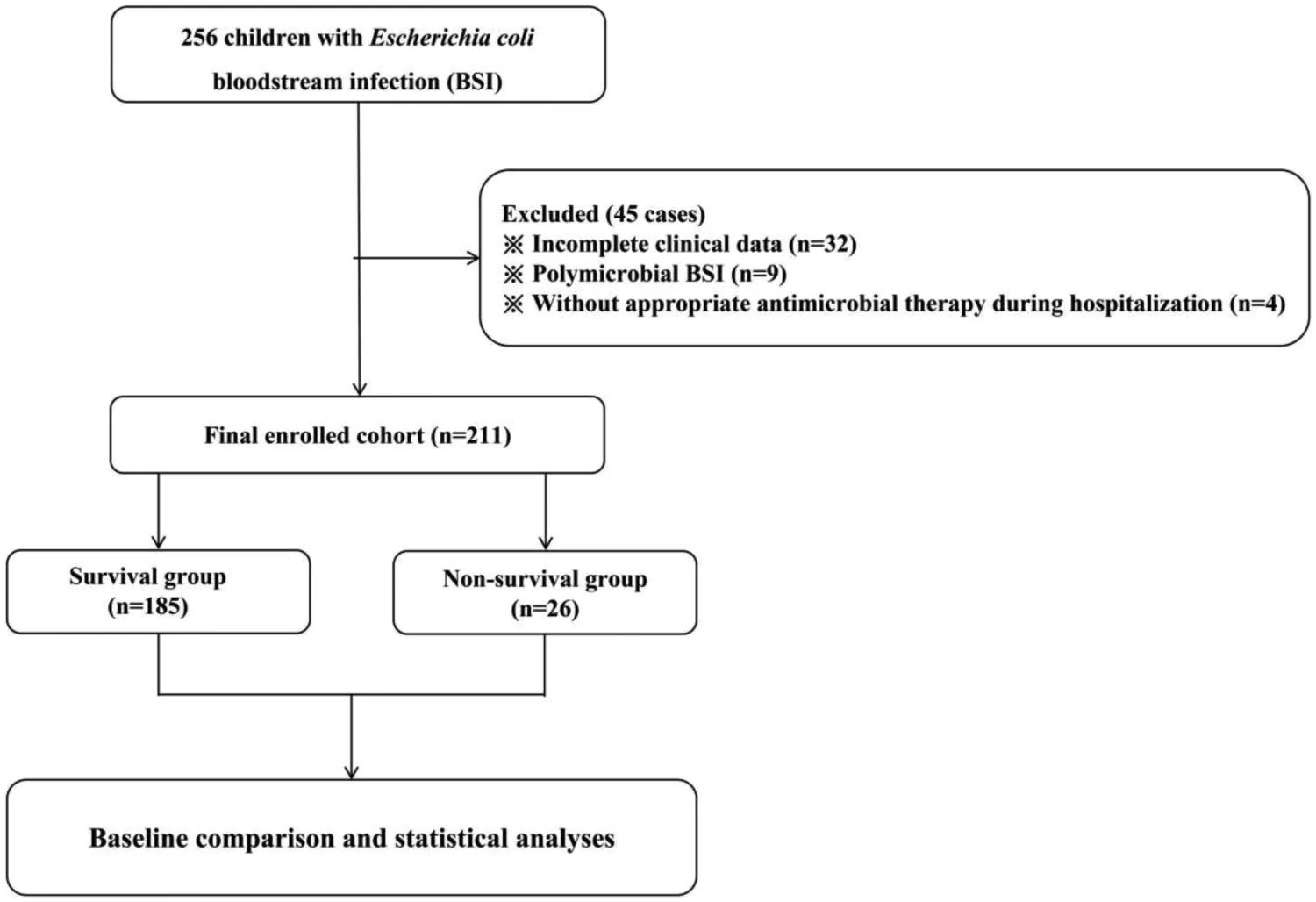
The flow chart of this study.

A total of 256 children with *E. coli* BSI were initially screened. Of these, 45 patients were excluded for the following reasons: incomplete clinical data (*n* = 32), polymicrobial BSI (*n* = 9), or lack of appropriate antimicrobial therapy during hospitalization (*n* = 4). The remaining 211 patients formed the final study cohort, which was divided into a survival group (*n* = 185) and a non-survival group (*n* = 26). Clinical characteristics were compared between the two groups. The patient selection process is illustrated in Figure 1.

### Baseline characteristics of 211 children with *Escherichia coli* BSI

The baseline characteristics of the entire cohort are shown in Table 1. The median age was 1.33 (IQR 0.19–8.46) years, and 60.19% were male. Leukemia was present in 41.23%, and chemotherapy-induced bone marrow suppression occurred in 45.97%. Hypoalbuminemia on admission was observed in 17.54%, and secondary hypoalbuminemia (developing after BSI onset) occurred in 10.90%. The median PRISM III score was 8.00 (IQR 0.00–9.00), and 9.00% required invasive mechanical ventilation. The incidence of septic shock was 8.53% and the in-hospital mortality was 12.32%.

**Table 1.** Characteristics of 211 children with Escherichia coli (*E. coli*) Bloodstream infection (BSI).

Characteristics	Number (%)/ Median (IQR)
Demographic data	
Age (years) (median, IQR)	1.33 (0.19–8.46)
Sex (male) (n, %)	127 (60.19%)
Admission characteristics	
With medical insurance (n, %)	118 (55.92%)
Emergency admission (n, %)	28 (13.27%)
History	
BSI history (n, %)	32 (15.17%)
Surgery history in 1 year (n, %)	17 (8.06%)
Underlying condition	
Bone marrow inhibition after chemotherapy (n, %)	97 (45.97%)
Chemotherapy in 1 month (n,%)	95 (45.02%)
Leukaemia (n, %)	87 (41.23%)
Hypoalbuminemia on admission (n, %)	37 (17.54%)
Congenital heart disease (n, %)	27 (12.80%)
Tumor (n, %)	7 (3.32%)
Malnutrition (n, %)	4 (1.90%)
Comorbidity	
Elevated of aminotransferase (n, %)	73 (34.60%)
Intracranial infection (n, %)	39 (18.48%)
Secondary hypoalbuminemia after BSI (n, %)	23 (10.90%)
Polyserous effusions (n, %)	12 (5.69%)
Laboratory parameters	
C-reactive protein (CRP) (mg/L) (median, IQR)	38.00 (15.00–67.00)
Procalcitonin (PCT) (ng/ml) (median, IQR)	2.40 (0.36–21.64)
Time to positivity (TTP (hours) (median, IQR)	15.07 (12.87–18.14)
Infection and microbiology	
Unknown source of BSI (n, %)	106 (50.24%)
Nosocomial infection (n, %)	86 (40.76%)
Extended spectyum beta lactamases (ESBLs) bacteria (n, %)	100 (47.39%)
Clinical severity	
Pediatric risk of mortality (PRISM) III scores (median, IQR)	8.00 (0.00–9.00)
Antimicrobial Therapy	
Susceptible antimicrobial agents before susceptibility tests (n, %)	207 (98.10%)
Susceptible antimicrobial agents before blood culture (n, %)	20 (9.48%)
Administrated with carbapenem plus vancomycin (n, %)	14 (6.64%)
Time to appropriate therapy (TTAT) (hours) (median, IQR)	2.13 (1.04–7.30)
Advanced therapy	
Invasive mechanical ventilation (n, %)	19 (9.00%)
Hemopurification (n, %)	7 (3.32%)
Length of hospitalization (days) (median, IQR)	20.25 (12.59–34.67)
Septic shock (n, %)	18 (8.53%)
In-hospital mortality (n, %)	26 (12.32%)

Note: Patients with hypoalbuminemia on admission were not classified as having secondary hypoalbuminemia; they were included in the non-secondary hypoalbuminemia reference group. Unknown source refers to the anatomical source of infection, while nosocomial infection refers to timing of acquisition (≥48 h after admission).

### Comparisons of clinical characteristics between survival and non-survival groups

As summarized in Table 2, non-survivors had significantly higher rates of hypoalbuminemia on admission (38.46% *vs*. 14.59%, *p* = 0.006), secondary hypoalbuminemia (34.62% *vs*. 7.57%, *p* < 0.001), invasive mechanical ventilation (61.54% *vs*. 1.62%, *p* < 0.001), septic shock (53.85% *vs*. 2.16%, *p* < 0.001), and polyserous effusions (23.08% *vs*. 3.24%, *p* = 0.001). They also had higher PRISM III scores (median 28.00 *vs*. 6.00, *p* < 0.001) and shorter TTP (median 12.96 *vs*. 15.50 h, *p* < 0.001).

**Table 2.** Comparisons of clinical characteristics between the survival group and non-survival group in 211 children with *E. coli* BSI.

Characteristics	Survival group (*n* = 185)	Non-survival group (*n* = 26)	P value
Demographic data			
Age (years) (median, IQR)	1.25 (0.19–7.42)	4.75 (0.40–11.31)	0.091
Sex (male) (n, %)	109 (58.91%)	18 (69.23%)	0.428
Admission characteristics			
With medical insurance (n, %)	105 (56.76%)	13 (50.00%)	0.661
Emergency admission (n, %)	21 (11.35%)	7 (26.92%)	0.056
History			
BSI history (n, %)	27 (14.59%)	5 (19.23%)	0.560
Surgery history in 1 year (n, %)	14 (7.57%)	3 (11.54%)	0.447
Underlying condition			
Bone marrow inhibition after chemotherapy (n, %)	84 (45.41%)	13 (50.00%)	0.818
Chemotherapy in 1 month (n,%)	82 (44.32%)	13 (50.00%)	0.738
Leukaemia (n, %)	77 (41.62%)	10 (38.46%)	0.925
Hypoalbuminemia on admission (n, %)	27 (14.59%)	10 (38.46%)	0.006*
Congenital heart disease (n, %)	26 (14.05%)	5 (19.23%)	0.552
Tumor (n, %)	5 (2.70%)	2 (7.69%)	0.208
Malnutrition (n, %)	4 (2.16%)	0 (0.00%)	1.000
Comorbidity			
Elevated of aminotransferase (n, %)	61 (32.97%)	12 (46.15%)	0.270
Intracranial infection (n, %)	34 (18.38%)	5 (19.23%)	1.000
Secondary hypoalbuminemia after BSI (n, %)	14 (7.57%)	9 (34.62%)	<0.001*
Polyserous effusions (n, %)	6 (3.24%)	6 (23.08%)	0.001*
Laboratory parameters			
CRP (mg/L) (median, IQR)	38.00 (15.00–65.00)	40.00 (16.25–87.25)	0.631
PCT (ng/ml) (median, IQR)	2.21 (0.34–18.10)	16.77 (1.33–93.03)	0.011*
TTP (hours) (median, IQR)	15.50 (13.08–18.63)	12.96 (10.77–14.45)	<0.001*
Infection and microbiology			
Unknown source of BSI (n, %)	98 (52.97%)	8 (30.77%)	0.056
Nosocomial infection (n, %)	75 (40.54%)	11 (42.31%)	1.000
ESBLs bacteria (n, %)	86 (46.49%)	14 (53.85%)	0.621
Clinical severity			
PRISM III scores (median, IQR)	6.00 (0.00–9.00)	28.00 (11.25–32.75)	<0.001*
Antimicrobial Therapy			
Susceptible antimicrobial agents before susceptibility tests (n, %)	182 (98.38%)	25 (96.15%)	0.411
Susceptible antimicrobial agents before blood culture (n, %)	18 (9.73%)	2 (7.69%)	1.000
Administrated with carbapenem plus vancomycin (n, %)	8 (4.32%)	6 (23.08%)	0.003*
TTAT (hours) (median, IQR)	2.08 (0.95–5.57)	5.36 (1.38–11.46)	0.189
Advanced therapy			
Invasive mechanical ventilation (n, %)	3 (1.62%)	16 (61.54%)	<0.001*
Hemopurification (n, %)	2 (1.08%)	5 (19.23%)	<0.001*
Length of hospitalization (days) (median, IQR)	22.00 (13.25–34.75)	9.48 (1.60–32.17)	0.006*
Septic shock (n, %)	4 (2.16%)	14 (53.85%)	<0.001*

*with statistical significance, *p* < 0.05. Note: Patients with hypoalbuminemia on admission were not classified as having secondary hypoalbuminemia; they were included in the non-secondary hypoalbuminemia reference group.

### Survival analysis

**Figure 2.**
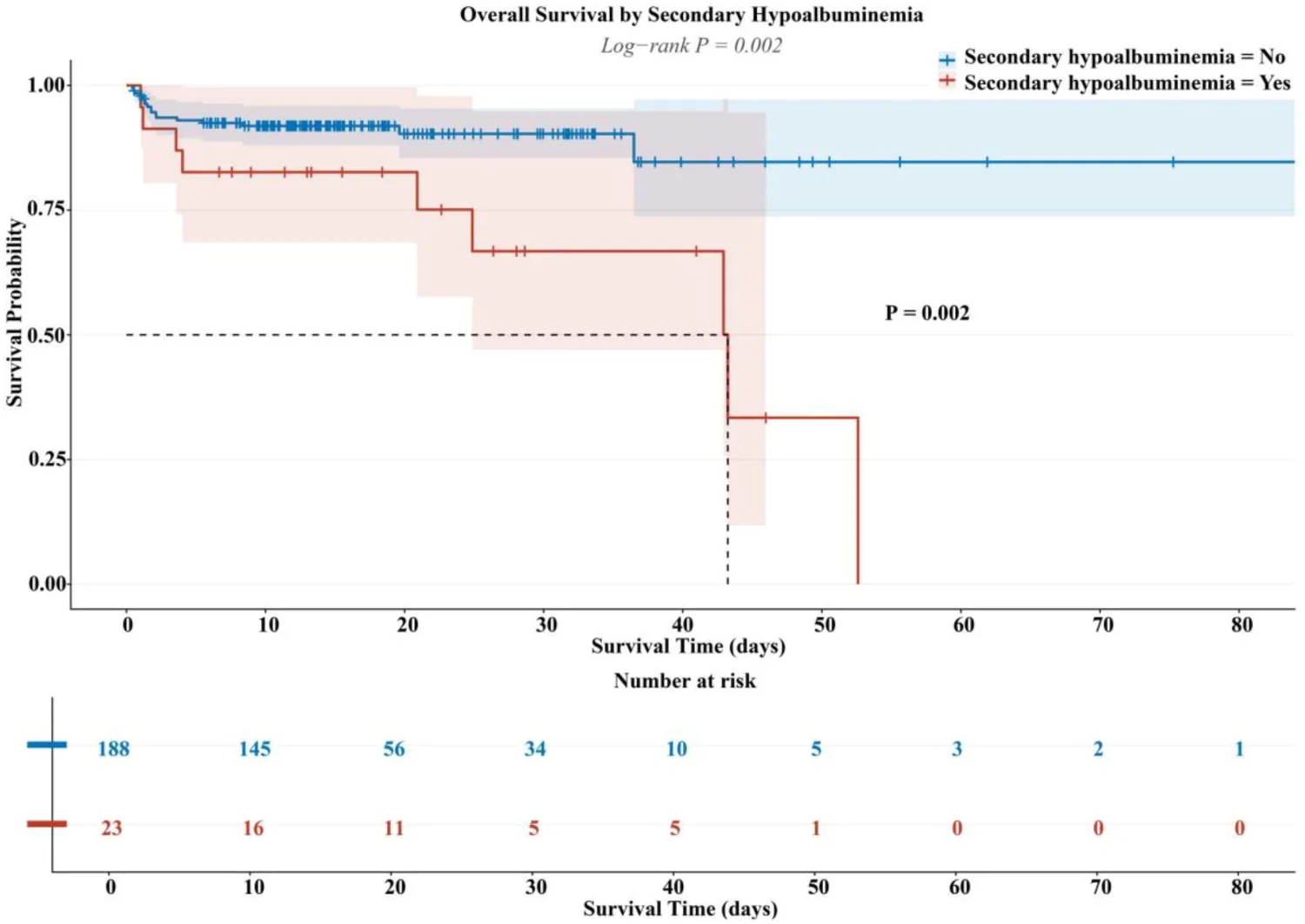
Kaplan-Meier survival curves for children with and without secondary hypoalbuminemia after *E. coli* BSI.

Kaplan-Meier curves showed that children who developed secondary hypoalbuminemia after BSI had significantly lower survival probability during hospitalization than those without secondary hypoalbuminemia (log-rank *p* = 0.002, Figure 2).

### Independent factors associated with in-hospital mortality

**Figure 3.**
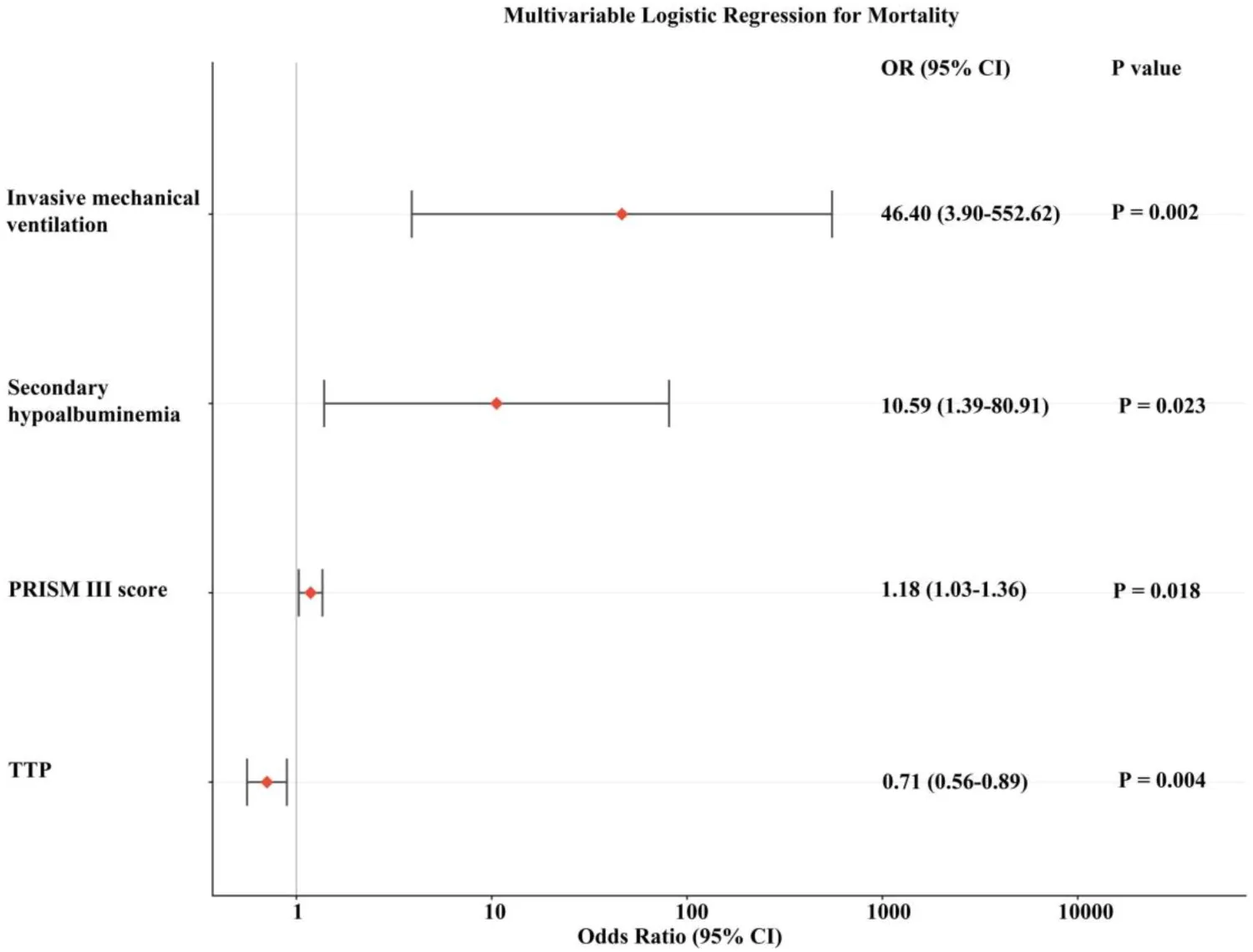
Multivariable logistic regression forest plot for mortality.

Univariate logistic regression results are shown in Table 3 (left columns) and Supplementary Fig. S1. After VIF assessment (all <5; Supplementary Fig. S2), 13 candidate variables were entered into the multivariable logistic regression model. The correlation matrix of these 13 variables is provided in Supplementary Fig. S3. Four factors remained independently associated with mortality (Table 3, right columns): secondary hypoalbuminemia (OR 10.59, 95%CI 1.39–80.91, *p* = 0.023), invasive mechanical ventilation (OR 46.40, 95%CI 3.90–552.62, *p* = 0.002), PRISM III score (OR 1.18, 95%CI 1.03–1.36, *p* = 0.018), and shorter TTP (OR 0.71 per hour increase, 95%CI 0.56–0.89, *p* = 0.004; i.e. each 1-hour longer TTP was associated with a 29% reduction in mortality odds). These are visualized in Figure 3. To assess whether PRISM III over-adjusted for disease severity, we performed a sensitivity analysis excluding it from the model. The association between secondary hypoalbuminemia and mortality remained significant (OR 12.23, 95% CI 1.94–77.17, *p* = 0.008; primary model: OR 10.59, 95% CI 1.39–80.91, *p* = 0.023) (Table S2, Fig. S4). To assess the discriminatory performance of the identified predictors, we performed ROC analysis based on a combined model incorporating the four independent predictors (invasive mechanical ventilation, secondary hypoalbuminemia, PRISM III score, and TTP). The combined model demonstrated high apparent discrimination for in-hospital mortality, with an AUC of 0.94 (95% CI: 0.88–1.00; Figure S5).

**Table 3.** Logistic regression analysis for risk factors of in-hospital mortality in 211 children with *E. coli* BSI.

Variables	Univariate analysis			Multivariate analysis		
	OR	95% CI	P	OR	95% CI	P
Invasive mechanical ventilation (n, %)	97.07	27.32-388.83	<0.001*	46.40	3.90–552.62	0.002*
Secondary hypoalbuminemia after BSI (n, %)	6.47	2.44-17.14	<0.001*	10.59	1.39–80.91	0.023*
PRISM III scores (median, IQR)	1.25	1.15-1.35	<0.001*	1.18	1.03–1.36	0.018*
TTP (hours)	0.76	0.65-0.89	<0.001*	0.71	0.56–0.89	0.004*
Septic shock (n, %)	52.79	15.04-185.26	<0.001*			
Hemopurification (n, %)	21.79	3.98-119.36	<0.001*			
Polyserous effusions (n, %)	8.95	2.64-30.39	<0.001*			
Administrated with carbapenem plus vancomycin (n, %)	6.64	2.09-21.07	0.001*			
Hypoalbuminemia on admission (n, %)	3.66	1.50-8.90	0.004*			
Emergency admission (n, %)	2.88	1.00-1.16	0.034*			
Age (years)	1.08	0.99-1.16	0.070			
PCT (ng/ml)	1.02	1.00-1.03	<0.001*			
Unknown source of BSI (n, %)	0.40	0.16-0.95	0.039*			

*with statistical significance, *p* < 0.05. Note: OR for TTP is expressed per hour increase; OR < 1 indicates that shorter TTP is associated with higher mortality.

### Sensitivity analysis (firth regression)

Given the limited number of death events (*n* = 26), Firth’s penalized logistic regression was performed as a sensitivity analysis to reduce small-sample bias and assess the robustness of the multivariable estimates. The results were consistent with the standard logistic model: secondary hypoalbuminemia (OR 8.02, 95 % CI 1.49–48.92, *p* = 0.016), invasive mechanical ventilation (OR 18.02, 95 % CI 2.73–156.66, *p* = 0.003), PRISM III score (OR 1.13, 95 % CI 1.02–1.28, *p* = 0.021), and TTP (OR 0.77, 95 % CI 0.62–0.92, *p* = 0.002) remained significantly associated with in-mortality (Supplementary Fig. S6).

### Subgroup and interaction analyses

Secondary hypoalbuminemia remained associated with mortality in both leukaemia (OR 10.05, 95%CI 2.41–41.95, *p* = 0.002) and non-leukaemia subgroups (OR 6.00, 95%CI 1.21–29.84, *p* = 0.029), with no significant interaction (*p* = 0.64). Similar results were seen for bone marrow suppression (P for interaction = 0.84). Subgroup Kaplan-Meier curves (Supplementary Fig. S7) and forest plot (Supplementary Fig. S8) are provided.

## Discussion

In this retrospective cohort study of 211 children with *E. coli* BSI, we identified four risk factors independently associated with in-hospital mortality: invasive mechanical ventilation, higher PRISM III score, shorter TTP, and - notably - secondary hypoalbuminemia (OR 10.59, 95% CI 1.39–80.91, *p* = 0.023). The association with secondary hypoalbuminemia remained robust in Firth’s penalised logistic regression (OR 8.02, 95% CI 1.49–48.92, *p* = 0.016) and across subgroups defined by leukemia and bone marrow suppression (interaction *p* > 0.6). These findings suggest that longitudinal monitoring of serum albumin may provide additional information for identifying children at increased risk of in-hospital mortality.

### Mechanical ventilation and PRISM III as predictors

The need for invasive mechanical ventilation was the strongest independent predictor of mortality (OR 46.40, 95% CI 3.90–552.62, *p* = 0.002). This is unsurprising, as mechanical ventilation reflects severe respiratory failure, often secondary to sepsis-induced acute respiratory distress syndrome (ARDS). Children requiring mechanical ventilation typically have profound physiological derangements, including impaired gas exchange, altered immune function, and increased susceptibility to ventilator-associated complications such as pneumonia and barotrauma [33]. Because invasive mechanical ventilation is generally reserved for patients with severe respiratory failure or multi-organ dysfunction, it is frequently used as a proxy measure of overall illness severity in critical care research [34]. Similarly, higher PRISM III scores (OR 1.18 per point, 95% CI 1.03–1.36, *p* = 0.018) confirmed that greater disease severity at admission strongly predicts mortality, consistent with extensive validation studies of PRISM III in pediatric critical care [6]. Both factors are well-established and reinforce the face validity of our model.

### Secondary hypoalbuminemia - a dynamic biomarker

Our findings extend the existing evidence on the prognostic role of albumin in pediatric sepsis. A large multicenter retrospective study (13 U.S. PICUs, 17,307 children) by Lee et al. [35] reported that early albumin infusion was associated with lower in-hospital mortality only in children with septic shock (OR 0.698, 95% CI 0.629–0.774, *p* < 0.001), but not in those without shock, underscoring the relevance of albumin status in the most severely ill patients. In our cohort, all children who developed hypoalbuminemia received albumin infusion as standard practice, though dosages were not recorded. Unlike these studies that focused on baseline or admission albumin, we specifically examined secondary hypoalbuminemia – hypoalbuminemia that develops after BSI onset in patients with normal pre-infection albumin levels. This distinction is clinically important, as secondary hypoalbuminemia reflects the acute host response to infection (e.g. capillary leakage, cytokine-mediated downregulation of albumin synthesis), rather than indicating pre-existing nutritional status [10,36]. Indeed, serum albumin is no longer considered a reliable marker of nutritional status [37], and the hypoalbuminemia observed in our cohort is most consistent with an inflammatory rather than a nutritional etiology. The prognostic value of albumin has also been explored through composite markers. The LAR has shown excellent discriminatory power for mortality in pediatric septic shock, and elevated LAR has been independently associated with acute kidney injury in septic patients (HR 1.49 per unit increase) [20]. In pediatric nosocomial infections, LAR at 24 h provided the highest predictive accuracy for mortality [21]. While these composite markers may offer incremental value, they require additional laboratory tests (lactate). Secondary hypoalbuminemia, in contrast, relies on a single routinely measured test, serum albumin, making it simpler and more accessible, particularly in resource-limited settings. Mechanistically, several pathways may explain the association between hypoalbuminemia and poor outcomes in sepsis. Low albumin has been shown to impair humoral immunity and neutrophil chemotaxis, increase pro-inflammatory cytokines (Interleukin-6, Tumor necrosis factor-alpha), and promote endothelial leakage, potentially leading to a vicious cycle of inflammation and organ dysfunction [10,38,39]. However, we acknowledge that these mechanisms are speculative in the context of our study, as we did not measure cytokines or endothelial function. Our data only support an association between secondary hypoalbuminemia and mortality, but do not establish a causal relationship. Further experimental and translational studies are needed to elucidate the underlying biology. While secondary hypoalbuminemia was independently associated with mortality in our multivariable model, we acknowledge that it likely represents a downstream consequence of severe infection, reflecting capillary leakage, systemic inflammation, and acute-phase response, rather than a primary biological cause of death. The independent association persisted after adjustment for PRISM III, a comprehensive severity score, but residual confounding cannot be excluded.

### Short TTP - the controversy

Short TTP independently predicted mortality in our study (OR 0.71 per hour increase, 95% CI 0.56–0.89, *p* = 0.004; i.e. each hour longer to positivity was associated with a 29% lower odds of death). This observation is supported by a large systematic review and meta-analysis of 24 studies, which identified short TTP as a robust marker of adverse clinical outcomes, being associated with nearly threefold higher mortality (OR 2.98, 95% CI 2.25–3.96) and more than fourfold increased odds of septic shock (OR 4.06, 95% CI 2.41–6.84) [40]. These findings suggest that shorter TTP reflects a greater pathogen burden and more severe systemic infection. However, a multicenter randomized controlled trial by Hamilton et al. (*n* = 3462, adults) found no association between TTP and mortality [41]. Several factors may explain this discrepancy. First, our cohort was exclusively pediatric with a single pathogen (*E. coli*), whereas the adult trial included mixed bacterial species. Second, we used an optimal TTP cut-off (consistent with meta-analyses demonstrating prognostic value), while Hamilton et al. [41] did not apply a cut-off. Third, TTP is commonly interpreted as a surrogate of bloodstream bacterial burden [8], although its relationship with mortality may vary across patient populations and clinical settings. Regarding TTAT, it was not an independent predictor in our model, likely because most patients received timely therapy (median TTAT 2.13 h, well below the 12-hour high-risk window reported by Van Heuverswyn et al. [42]).

### Comparison with other albumin-related markers

Recent studies have identified several albumin-based composite markers with strong predictive value. Özdemir et al. [43] compared the prognostic utility of blood urea nitrogen/albumin, lactate/albumin, and CRP/albumin ratios in 147 critically ill geriatric patients requiring inotropic support in the emergency department, finding that all three ratios independently predicted 30-day mortality; the lactate/albumin ratio showed the strongest prognostic value (AUC 0.85). For instance, the ferritin-albumin ratio has been shown to be an excellent prognostic marker for mortality in children with sepsis, with an area under the ROC curve approaching 1.00 at optimal cut-off values [15]. The blood urea nitrogen-to-albumin ratio independently predicts 30-day mortality and severity in patients with *E. coli* bacteraemia [14]. A multi-center PICU study of 1,329 children reported that admission hypoalbuminemia was a strong predictor of mortality (OR 0.32, 95% CI 0.26–0.40), with a predictive rate of 84.5% [18]. Turcato et al. also found that serum albumin at Emergency department admission independently predicted 30-day mortality in 962 infected patients (adjusted HR 3.77, 95% CI 2.19–6.44) [19]. While these composite markers may offer incremental predictive value, they require additional laboratory tests. Secondary hypoalbuminemia, in contrast, relies on a single, routinely measured, inexpensive test (serum albumin), making it particularly suitable for resource-limited settings where complex scoring systems or specialized biomarkers are not available.

### Limitations

This single-center retrospective study included a modest sample size and only 26 death events, limiting statistical precision, generalizability and the complexity of the multivariable model. Although Firth’s penalized logistic regression was used to reduce small-sample bias, it does not eliminate the possibility of model overfitting. Additionally, our cohort was enriched with children with leukemia (41.23%) and chemotherapy-induced bone marrow suppression (45.97%), reflecting the patient population at our tertiary oncology referral center. Consequently, our findings may not be generalizable to immunocompetent children or community-based settings. Second, the definition of secondary hypoalbuminemia may introduce immortal time bias, as patients must survive long enough to develop hypoalbuminemia and be classified as exposed. Landmark analyses at Days 7 and 14 yielded consistent associations with in-hospital mortality (HR 5.09 and 6.12, both *p* < 0.01), with no significant change over time (P for interaction = 0.088). (Table S1, Fig. S9). Attenuation at later time points likely reflects survivor effects and reduced power (Table S1). Third, we lacked data on other severity indicators such as vasopressor requirement, lactate, or organ dysfunction scores. However, we included PRISM III, a validated composite severity score, and a sensitivity analysis excluding it confirmed the robustness of the association (Supplementary Table S2). Nevertheless, residual confounding by unmeasured severity indicators cannot be excluded. Fourth, TTP thresholds may vary across hospitals, but standardized blood culture procedures can reduce this variability. Fifth, the narrow distribution of TTAT (median 2.13 h) may have compressed its influence in multivariable analysis. Sixth, because albumin measurements were performed according to clinical need, the frequency of assessment was not uniform across patients, which may have resulted in differential detection of secondary hypoalbuminemia. Seventh, we did not collect data on the dosage of albumin administered to patients who developed hypoalbuminemia, and thus cannot assess the potential impact of albumin replacement therapy on outcomes. Finally, the observational design precludes causal inference, our study only identifies an association, not a causal relationship.

## Conclusions

Secondary hypoalbuminemia that develops after *E. coli* BSI was independently associated with increased in-hospital mortality in children. These findings suggest that secondary hypoalbuminemia may serve as a dynamic prognostic marker for early risk stratification. Prospective multicenter studies with external validation are needed to determine its clinical utility.

## Supplementary Material

Supplementary_clean.docx

## Data Availability

Data from this study can be obtained from the corresponding author with rational request.
