# Supplementary material for: Secondary hypoalbuminemia is independently associated with in-hospital mortality in children with Escherichia coli bloodstream infection
Source: Ann Med. 2026 Sep 10;58(1):2731669. doi: 10.1080/07853890.2026.2731669 (PMC13564163; doi:10.1080/07853890.2026.2731669)
Supplement: Supplementary file 1 [file IANN_A_2731669_SM1104.docx]

**Table S1 Full landmark analysis results at Days 7, 14, 21, and 28.**

| Landmark Time | No. at Risk | No. of Events | OR (95% CI) | OR P | HR (95% CI) | HR P |
| --- | --- | --- | --- | --- | --- | --- |
| 7 days | 191 | 13 | 18.74 (5.41-65.00) | <0.001 | 5.09 (1.59-16.30) | 0.006* |
| 14 days | 138 | 12 | 17.39 (4.60-65.76) | <0.001 | 6.12 (1.76-21.24) | 0.004* |
| 21 days | 104 | 8 | 10.64 (2.27-49.94) | 0.003 | 3.76 (0.84-16.85) | 0.084 |
| 28 days | 82 | 7 | 8.67 (1.68-44.60) | 0.010 | 2.61 (0.54-12.65) | 0.232 |

*with statistical significance, P <0.05. Abbreviations: BSI, bloodstream infection; CI, confidence interval; HR, hazard ratio; OR, odds ratio. Conditional on survival to each landmark time; time axis for HR = remaining length of stay after landmark.

**Table S2 Sensitivity analysis excluding PRISM III**

| Model | OR | 95% CI | P |
| --- | --- | --- | --- |
| Full model (with PRISM III) | 10.59 | 1.39-80.91 | 0.023* |
| Sensitivity (without PRISM III) | 12.23 | 1.94-77.17 | 0.008* |
| Full model with Firth correction | 8.02 | 1.49-48.92 | 0.016* |
| Sensitivity with Firth correction | 9.17 | 1.87-47.98 | 0.008* |

*with statistical significance, P <0.05. Abbreviations: OR, odds ratio; CI, confidence interval.

**Fig. S1. Univariate logistic regression forest plot (all variables).**

**Fig. S2 Variance inflation factor (VIF) bar plot (pre-model multicollinearity assessment).**

**Fig. S3 Correlation heatmap of the candidate 13 variables (after VIF correction).**

**Fig. S4. Forest plot comparing ORs with/without PRISM III**

**Fig. S5 ROC curve of the combined model for predicting in-hospital mortality.**

**Fig. S6 Comparison of standard maximum likelihood estimation(MLE) *vs*. Firth penalised logistic (PL) regression (forest plot).**

**Fig. S7 Kaplan‑Meier curves for survival according to secondary hypoalbuminemia, stratified by leukaemia (a,b) and bone marrow suppression (c,d).**

**Fig. S8 Subgroup forest plot for secondary hypoalbuminemia.**

**Fig. S9 Time-varying hazard ratio curve for secondary hypoalbuminemia across length of stay.**
